# Quality prediction and classification of resistance spot weld using artificial neural network with open-sourced, self-executable and GUI-based application tool *Q-Check*

**DOI:** 10.1038/s41598-023-29906-0

**Published:** 2023-02-21

**Authors:** Suhaila Abd Halim, Yupiter H. P. Manurung, Muhamad Aiman Raziq, Cheng Yee Low, Muhammad Saufy Rohmad, John R. C. Dizon, Vladimir S. Kachinskyi

**Affiliations:** 1 Smart Manufacturing Research Institute (SMRI), Universiti Teknologi MARA (UiTM), 40450 Shah Alam, Selangor Malaysia; 2 Faculty of Computer and Mathematical Sciences, Universiti Teknologi MARA (UiTM), 40450 Shah Alam, Selangor Malaysia; 3 School of Mechanical Engineering, Universiti Teknologi MARA (UiTM), 40450 Shah Alam, Selangor Malaysia; 4 Innovationlabs.my, Universiti Tun Hussein Onn (UTHM), Batu Pahat, Malaysia; 5 School of Electrical Engineering, Universiti Teknologi MARA (UiTM), 40450 Shah Alam, Selangor Malaysia; 6 Design, Research, Extension in Additive Manufacturing, Advanced Manufacturing (DR3AM) Center, Bataan Peninsula State University, City of Belanga, Philippines; 7 E.O. Paton Electric Welding Institute, Kiev, Ukraine

**Keywords:** Mechanical engineering, Computational science

## Abstract

Optimizing Resistance spot welding, often used as a time and cost-effective process in many industrial sectors, is very time-consuming due to the obscurity inherent within process with numerous interconnected welding parameters. Small changes in values will give effect to the quality of welds which actually can be easily analysed using application tool. Unfortunately, existing software to optimize the parameters are expensive, licensed and inflexible which makes small industries and research centres refused to acquire. In this study, application tool using open-sourced and customized algorithm based on artificial neural networks (ANN) was developed to enable better, fast, cheap and practical predictions of major parameters such as welding time, current and electrode force on tensile shear load bearing capacity (TSLBC) and weld quality classifications (WQC). A supervised learning algorithm implemented in standard backpropagation neural network gradient descent (GD), stochastic gradient descent (SGD) and Levenberg–Marquardt (LM) was constructed using TensorFlow with Spyder IDE in python language. All the display and calculation processes are developed and compiled in the form of application tool of graphical user interface (GUI). Results showed that this low-cost application tool Q-Check based on ANN models can predict with 80% training and 20% test set on TSLBC with an accuracy of 87.220%, 92.865% and 93.670% for GD, SGD and LM algorithms respectively while on WQC 62.5% for GD and 75% for both SGD and LM. It is also expected that tool with flexible GUI can be widely used and enhanced by practitioner with minimum knowledge in the domain.

## Introduction

Resistance spot welding (RSW) is widely employed in manufacturing sectors for sheet metal joining because of its high speed and scalability to automation in high-volume, high-rate production. Additionally, RSW is a type of resistance welding that is frequently utilised in place of rivets, screws, soldering, and brazing on a range of goods. The approach is extensively used to combine components made of low carbon steel. High-strength low- alloy steel, stainless steel, nickel, aluminium, titanium and copper alloys are also spot welded commercially^1^.

Two or more metal sheets are bonded together using resistance spot welding by putting an electric current across them. The current is transmitted through electrodes that are applied onto the metal surfaces of the components to be welded. The heat generated by the running current melts the metal, resulting in the formation of a welding spot. The amplitude and length of the current influence the amount of heat generated at the location. The current and duration are precisely regulated and matched to the material, thickness, and kind of electrodes. As with any other type of welding, the quality of the joint is specifically correlated to the welding input parameters. A common problem is choosing and implementing the process input parameters to achieve a well-welded joint with the appropriate strength^2^.

The problem of choosing the optimal input parameters gives motivation to this study. The objectives related to this study are to develop ANN algorithm and GUI-based application tool to predict the parameters of RSW. The ANN consists of mathematical structures that emulate the biological nervous system's behaviour. It maps non-linear and dynamic designs with parallel, distributed, and adaptive computation^3^.

Hamedi et al.^4^ standardized three major processes of input parameters: welding current, welding time, and electrode force for spot welding of the body parts. The influence of these parameters on sub-assembly deformation were tested experimentally. Neural networks and multi-objective genetic algorithms were applied to select the optimal welding parameter values that generated the least dimensional variance values in the sub-assemblies.

The ANN can also be referred as a multilayer perceptron (MLP). According to Haruna Chiroma et al.^5^, there are many learning algorithms in MLP. One commonly used is the backpropagation algorithm or the backpropagation neural network (BPNN). However, in BPNN, the GD algorithm requires extended time to converge. Thus, the Levenberg–Marquardt (LM) algorithm is used for accelerating convergence^6^. The supervised learning algorithms used in this study are the BPNN or also known as standard backpropagation neural network algorithm which uses the GD, stochastic gradient descent (SGD), and the LM algorithm. Numerous past research used BPNN to optimize parameter, predict fatigue life and predict weld quality of RSW^6–12^. A graphical user interface (GUI) is used to make the ANN algorithm in graphical form that is easy to use by end user to make predictions^13,14^. A user friendly and easy to use GUI is beneficial to be developed as to effectively deal with the developed ANN algorithm.

In this study, the BPNN algorithms are implemented to optimise the parameters in RSW. These techniques are adapted for the prediction of TSLBC value and classification of quality for resistance spot welding. It is followed by developing an application tool consisting of several GUIs that simplify the computer environment. The tool can be used by other researcher and industry practitioner which enable them to easily use and gather experiment results using the developed tool without prior knowledge in the domain.

## Experimental procedure and data collection

The experimental investigation stem of this paper is from the previous literature^3^. The chemical composition of the austenitic stainless steel SS304 sheets includes material properties, equipment, test specimen and RSW experimental input and output parameters. The chemical composition of the 304 ASS sheets is shown in Table 1.

**Table 1:** Chemical composition of SS304 (wt%).

C	Cr	Ni	Si	Mn	Mo	Al	Co
0.08	18.03	8.74	0.426	1.153	0.36	0.003	0.17

Cu	Nb	Ti	V	W	Fe	P	S
0.39	0.02	0.004	0.05	0.03	70.48	0.019	0.002

The SS304 sheets were welded in 50 Hz single-phase equipment with water-cooled truncated cone RWMA Group A Class 2 electrodes with a face diameter of 4.5 mm. The ultrasonic spot-welding research transducer combines a captive water column delay and a replaceable rubber membrane to ensure a secure coupling to the weld surfaces. The transducer was 20 MHz in frequency and 4.5 mm in diameter. The sheet measured 0.8 mm thickness in diameter. The physical properties of the SS304 sheets that consisted of yield strength, tensile strength, total elongation, and microhardness are summarized in Table 2.

**Table 2:** Mechanical properties of SS304.

Yield strength (MPa)	Tensile strength (MPa)	Total elongation (%)	Microhardness (HV, 100 g)
290	675	70	162

**Figure 1:**
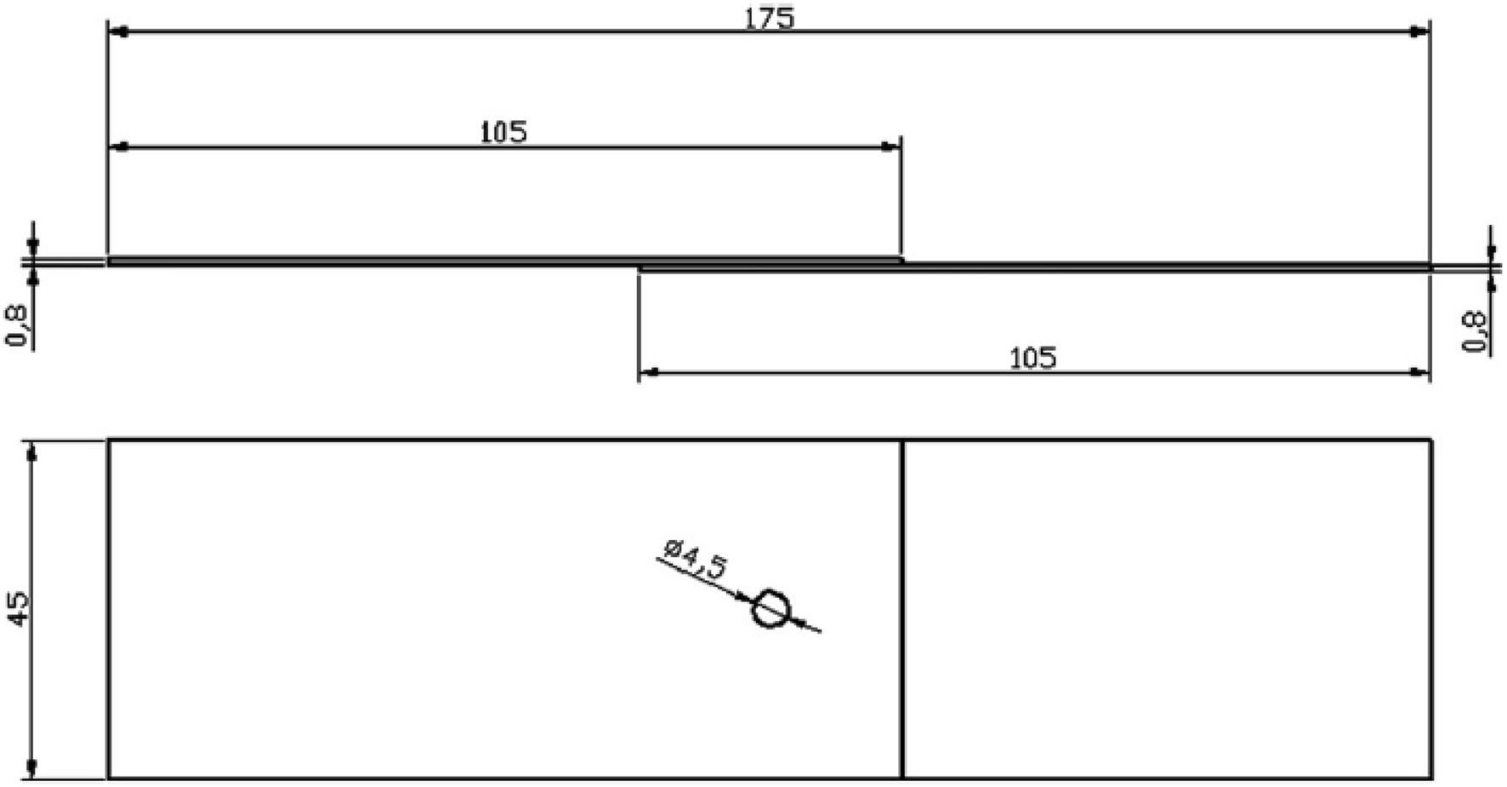
Dimensions of spot-welded tensile shear test specimens (mm).

The RSW process employed a weld interval between 12 and 2 cycles, with a one-cycle step decrement. The welding current varied from 6.5 to 1.5 kA RMS, with a phase decreased of 0.5kA RMS and two electrode force values of 1000 N and 1500 N. Tensile shear specimens were prepared in accordance to ISO 14273. The dimension of test specimen is shown in Fig. 1.

Based on RSW dataset extracted from^3^, there were 36 data available with three inputs as corresponding outputs of TSLBV value (kN) and the quality level. The three input parameters were weld time (cycle), weld current (kA) and electrode force (N). The three parameters are selected because they are the important parameters in RSW^15^ The class value was either Bad, Good, or Worst with a TSLBC Value for each data point. Table 3 shows the used dataset of RSW.

**Table 3:** Data Collection of RSW dataset.

Weld time (cycle)	Weld current (kA)	Electrode force (N)	TSLBC value (kN)	Quality level
7	6.16	1500	6.91	Good
12	5.64	1500	6.36	Good
3	5.51	1500	5.5	Bad
5	4.98	1500	5.75	Bad
8	4.46	1500	5.8	Bad
9	3.93	1500	5.14	Bad
3	4.06	1500	4.01	Bad
3	3.54	1500	3.58	Bad
9	2.88	1500	4.49	Bad
12	6.56	1500	6.5	Good
3	6.56	1500	5.55	Bad
8	2.49	1500	3.76	Bad
4	2.49	1500	2.95	Bad
12	1.96	1500	3.42	Bad
11	6.56	1000	6.78	Good
7	6.42	1000	6.68	Good
9	5.9	1000	6.38	Good
12	5.51	1000	6.06	Good
5	5.64	1000	6.28	Good
9	4.98	1000	6.11	Good
6	4.98	1000	5.92	Bad
12	4.59	1000	5.92	Bad
7	4.46	1000	5.98	Good
11	3.93	1000	5.17	Bad
8	3.93	1000	5.56	Bad
4	4.06	1000	4.67	Bad
10	3.41	1000	4.93	Bad
4	3.54	1000	4.21	Bad
7	3.01	1000	4.21	Bad
11	1.96	1000	3.63	Bad
3	1.96	1000	2.29	Bad
10	2.49	1000	3.18	Bad
2	2.49	1000	2.7	Bad
5	1.57	1000	1.67	Worst
11	1.7	1500	2.1	Bad
3	1.44	1500	1.36	Worst

Based on literature^3^, the quality level was divided into good, bad and worst depending on the TSLBC value. The quality level of the RSW joints was classified based on the TSLBC value as shown on Table 4.

**Table 4:** The quality level classification of RSW joint.

TSLBC of the RSW joint	Quality level
5.93 kN ≤ TSLBC	Good
1.83 kN ≤ TSLBC < 5.93 kN	Bad
TSLBC < 1.89 kN	Worst

The classification of the quality level of RSW joint considered in good condition if the TSLBC values are greater than or equal to 5.93 kN. The quality level is bad if the TSLBC is less than 5.93 kN or greater than or equal to 1.83 kN. It considered worst if the values are less than 1.89 kN.

In this study, ANN was applied to predict the TSLBC value using linear regression while multiclassification was used to classify the quality level.

## Prediction and classification using artificial neural network

The ANNs are mathematical models that emulate the behaviour of the human nervous system and capable of parallel, distributed, and adaptive computation which enables them to map non-linear and complex structures^16^. The ANN consists of a set of connected units generally known as neurons or nodes. The neurons are arranged in a layer, whereby the signal is transmitted between one neuron and another in each connection. The receiving neuron processes the signal and signals it to its associated downstream neurons within the network.

There are three main types of ANN layers: input, hidden, and output. The input layer receives different information forms, and the data flow from the input layer to the hidden layer to the output layer. Most neural networks are completely interconnected between layers, and the links are weighted. The higher the weight number, the greater the effect of one unit on another.

**Figure 2:**
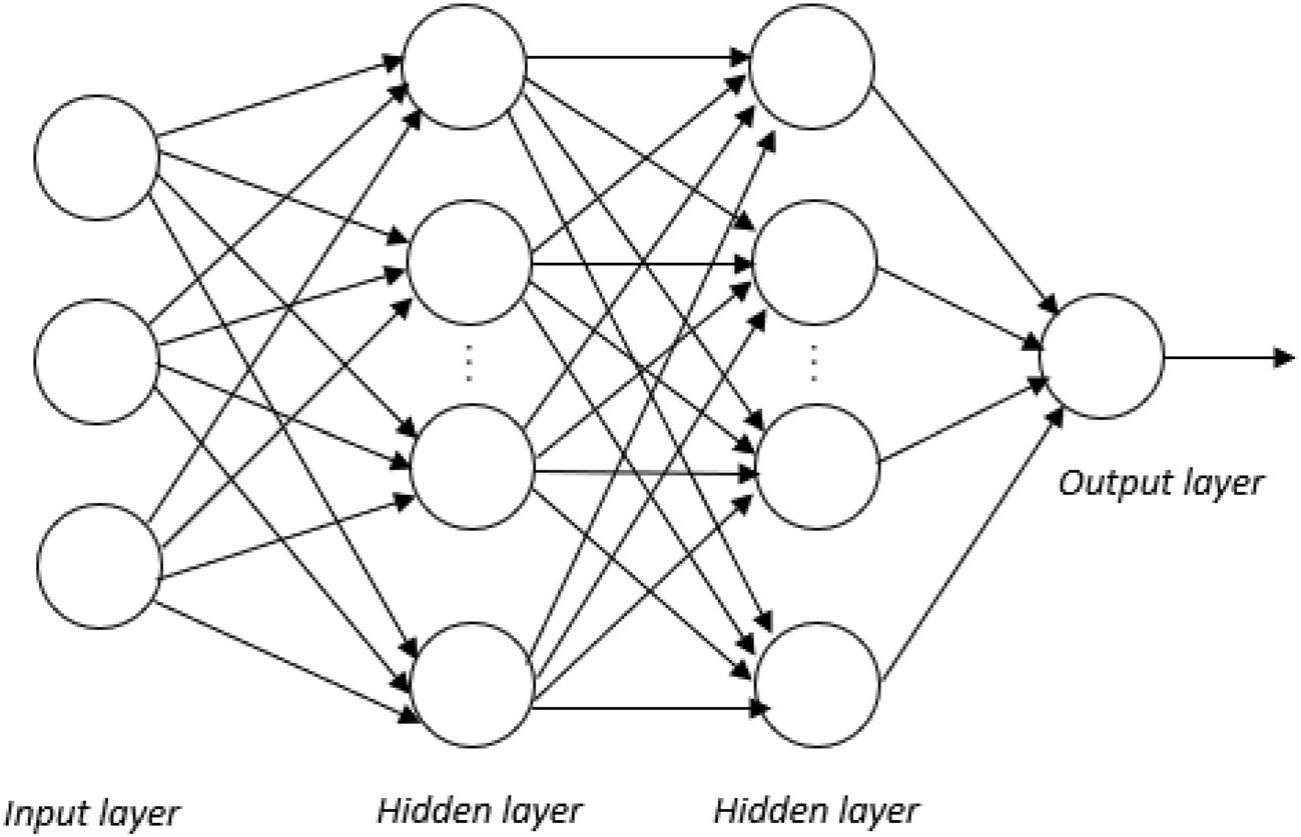
Multilayer perceptron feed-forward network.

Multilayer perceptron feed-forward ANN is used to solve complex predictive modelling problems. They usually have an input layer, one or two hidden layers, and a single output layer. Neurons are computational units in the hidden layers that conduct non-linear mapping between inputs and outputs. This study focuses on multilayer perceptron neural network. It takes a set of 3 input values which are electrode force (EF), welding current (WC), and welding time (WT). The prediction on the numerical output is TSLBC and categorical output is the weld quality: worst, bad, good. Figure 2 shows the multilayer perceptron feed-forward network used in predicting TSLBC of RSW.

### Optimizer and training algorithm for back propagation neural network

Training a multilayer perceptron is the process of determining the individual weights' values so that the network can correctly overcome the relationship for modelling. Like other probabilistic neural networks, this neural network needs only a fraction of the training samples. The BPNN is commonly applied to multilayer feed-forward ANN due to its ability to "learn" system properties via nonlinear mapping^17^.

The BPNN is an algorithm using gradient descents to measure the loss function. The gradient function can be expressed as Eq. (1).

1 $$ \frac{d}{{d\theta_{j} }}J\left( \theta \right) = - \frac{2}{n}\sum\limits_{i = 1}^{n} {(y_{t} - y_{pred} )\,f^{\prime}\left( {\sum\limits_{i = 1}^{k} {w_{j} x_{i}^{l} + b} } \right)\,x_{{w_{j} }}^{l} } $$

$\frac{d}{{d\theta_{j} }}J\left( \theta \right)$ = the gradient/derivative of the objective function J with respect to jth weight and bias, $l$ = layer ($l$ = 0 is input layer), $w_{j}$ = jth weight, $x_{{w_{j} }}^{l}$ = input of data at jth weight for $l$th layer, $y_{t}$ = true value, $y_{pred}$ = predicted value, $n$ = Number of datapoint, $x_{i}^{l}$ = Input data at ith input for $l$th layer, $k$ = Total neuron at certain layer.

The SGD is also a gradient descent type, using 1 example cost gradient at each iteration instead of using the sum of ALL examples cost gradient. It regularly updates the model parameter. The benefit of the algorithm is to update model parameters so that it converges in less time and needs less memory as the loss values do not need to be stored^18^. The gradient function for SGD can be expressed as Eq. (2).

2 $$ \frac{d}{{d\theta_{j} }}J\left( \theta \right) = - 2(y_{t} - y_{pred} )f^{\prime}\left( \sum \right)x_{{w_{j} }}^{\,l} $$

An analytical parameter used to evaluate the network's performance is an objective function that refers to loss function. Loss is defined as the difference between the expected and actual performance values. The ultimate goal is to eliminate error to maximize the accuracy of the model's forecasts. Mean square error (MSE) and cross-entropy are two objective functions commonly used.

Mean squared error (*MSE*) is one of the mostly used objective functions, measured as the average squared difference between predictions and actual values. It is only concerned with the average magnitude of error, irrespective of its direction. The *MSE* can be represented as Eq. (3)^19^.

3 $$ MSE = \frac{1}{N}\sum\limits_{n} {(y_{t} - } y_{pred} )^{2} $$

where $N$ is a number of output nodes or neurons.

Categorical cross-entropy is often called Softmax loss activation function plus cross-entropy loss. The categorical entropy objective function is used when there are two or more label classes. The categorical cross-entropy can be represented as Eq. (4)^20^.

4 $$ y_{{pred_{j} }} = \frac{{e^{{\left( {x_{{w_{j} }}^{l} } \right)}} }}{{\sum\limits_{i = 1}^{k} {e^{{\left( {x_{i}^{l} } \right)}} } }}\quad CE = - \frac{1}{n}\sum\limits_{i = 1}^{C} {y_{t} \log \left( {y_{pred} } \right)} {\kern 1pt} $$

Weight is used to minimize the prediction error. After the gradient of the objective function achieves global minima, it will maximize the objective function. Hence, the weight needs to be updated. It is a process where the current weight is subtracted with the gradient of the objective function multiplied by the learning rate. The update rule of BPNN is expressed as Eq. (5)

5 $$ w_{j + 1} = w_{j} - n\frac{d}{{dw_{j} }}J\left( w \right)\quad b_{j + 1} = b_{j} - n\frac{d}{{db_{j} }}J\left( b \right) $$

$w_{j}$ is a current weight in a neural network. $w_{j + 1}$ is a new weight in a neural network. $b_{j}$ is a current bias weight in a neural network. $b_{j + 1}$ is a new bias weight in a neural network. $\eta$ is a learning rate.

Another well-known optimizer is the Levenberg Marquardt (LM) which is the combination of a gradient descent method and the Gauss–Newton method designed to minimize a non-linear function^21^. In ANN, LM is suitable for training small and medium-sized problems. The Gauss–Newton approximates the Hessian matrix, *H* expressed as Eq. (6).

6 $$ H = J^{T} J $$

where *J* is the Jacobian matrix, and the LM introduces another approximation to the Hessian matrix as expressed in Eq. (7)^22^.

7 $$ H = J^{T} J + \mu I $$

where $\mu$ is a damping parameter which is always a positive number, and *I* is the identity matrix. The update rule of LM is expressed as Eq. (8).

8 $$ w_{j + 1} = w_{j} - \left[ {J^{T} \left( {w_{j} } \right)J\left( {w_{j} } \right) + \mu_{j} I} \right]^{ - 1} J^{T} \left( {w_{j} } \right)v\left( {w_{j} } \right) $$

where $w_{j + 1}$ is a new weight calculated as gradient function, $w_{j}$ is the current weight using the Newton algorithm, *j* is the iteration index, $\mu_{j}$ is a damping parameter and $v$ is an error vector.

**Figure 3:**
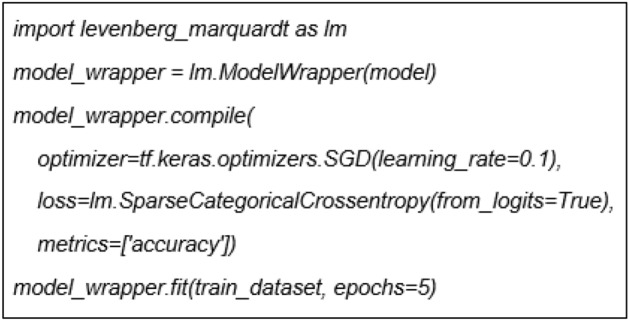
Implemented LM algorithm using Python.

Differ from GD and SGD which are available in Keras libraries, the Python code for LM algorithm is developed and embedded in the application tool. The LM algorithm implemented in ANN models originated from tf.keras.Model. Referring to the lines of codes in Fig. 3, the process is comparable on how the models are often trained in Keras. The distinction is that the fit function is invoked on a ModelWrapper class rather than on the model class directly.

As the algorithm shown, the LM optimizer is an advancement of GD and Newton method which can be seen in the separately self-developed model known as *model_wrapper.* This implementation of the LM algorithm refers to the previous work of^23^*.*

The LM approaches gradient descent when the $\mu$ is very large, and it approaches the Gauss–Newton algorithm when it $\mu$ is very small or nearly zero. The LM is fast, stable, and more robust than the Gauss–Newton as it converges well. But it is a bit slower than Gauss–Newton. The LM converges faster than the gradient descent method, and it requires much memory compared to Gauss–Newton and gradient descent^6^.

### Activation functions and ANN structure

Activation function, also known as transfer function, is used in neural network to calculate and update the weighted sum and bias.

The activation function for the hidden layers is the Rectified linear units (ReLu) function. The ReLu activation function can be defined as Eq. (9).

9 $$ f\left( x \right) = \left\{ {\begin{array}{*{20}c} 0 & {{\text{for}}\,x < 0} \\ x & {{\text{for}}\,x \ge 0} \\ \end{array} } \right. $$

There are two types of activation functions used in this paper for the output layer since there are two different types of output from the dataset, namely scalar for TSLBC and categorical variable for weld quality. The activation function used to predict the TSLBC of RSW is the Linear activation function as defined in Eq. (10).

10 $$ f(x) = mx $$

In addition, the activation function used to predict the quality of weld is Softmax as defined in Eq. (11).

11 $$ f\left( {x_{i} } \right) = \frac{{e^{{x_{\,i} }} }}{{\sum\limits_{j = 1}^{k} {e^{{x_{\,j} }} } }} $$

where $x_{i}$ the ith element of input values and $j$ is the total number of input values.

Different activation functions are applied for both hidden and output layers due to their capability and type of prediction.

Table 5 summarises the activation functions of the ANN model used in this study. For both models, ReLu is applied in hidden layer. Linear and Softmax are used in output layer as activation function for linear regression and multi-classification, respectively.

**Table 5:** Activation function used for the ANN model.

Type of model	Hidden layer	Output layer
Linear regression	ReLu	Linear
Multi-classification	ReLu	Softmax

The ANN inputs are vectors with three components, one for each RSW parameter of WT, WC, and EF which are used to predict TSLBC and weld quality. Hence, the ANN input layer contains three neurons that represent the three inputs. A supervised learning mechanism is used to learn from the labelled training data. This indicates that each input must be followed by a target. This target is numerical and categorical which contain the value of TSLBC and welding quality of Good, Bad, and Worst obtained using the respective input's welding parameters. As a result, the output layer of the ANN contains one and three neurons for TSLBC value and welding quality, respectively. The number of hidden layers used in this study are in linear regression and multi-classification, while the number of neurons inside the hidden layer is 39 by using the proposed model as suggested by Pashazadeh et al.^12^. The suggested model by^24^ produced minimal error as compared with the other models. Table 6 summarizes the ANN structures used in this study.

**Table 6:** ANN structure for numerical and categorical output.

Type of model	Numerical output	Categorical output
Linear regression	3-39-1	3-39-3
Multi-classification	3-39-39-1	3-39-39-3

According to Basheer and Hajmeer^25^ one hidden layer is enough to get a proper prediction when database is small. Hence only one hidden layer is applied for linear regression for both numerical and categorical output.

Referring to Table 3, the entire data set namely 36 input/target pairs corresponding to the 36 RSW data is randomly split into two subsets which are:

- *Training subset* (80%) with 26 input/target pairs for training the ANN. The synaptic weights (each link between neurons is associated with a synaptic weight) are modified periodically to minimize the error between the experimental outputs and their respective targets.
- *Testing subset* (20%) with 8 input/target pairs to evaluate the accuracy of the ANN after the training process.

80% is the best division of training and testing subset according to Gholamy et al.^26^. The whole processes in ANN algorithms are combined to develop an application tool that consists of several GUIs.

## Application GUI development of Q-Check

**Figure 4:**
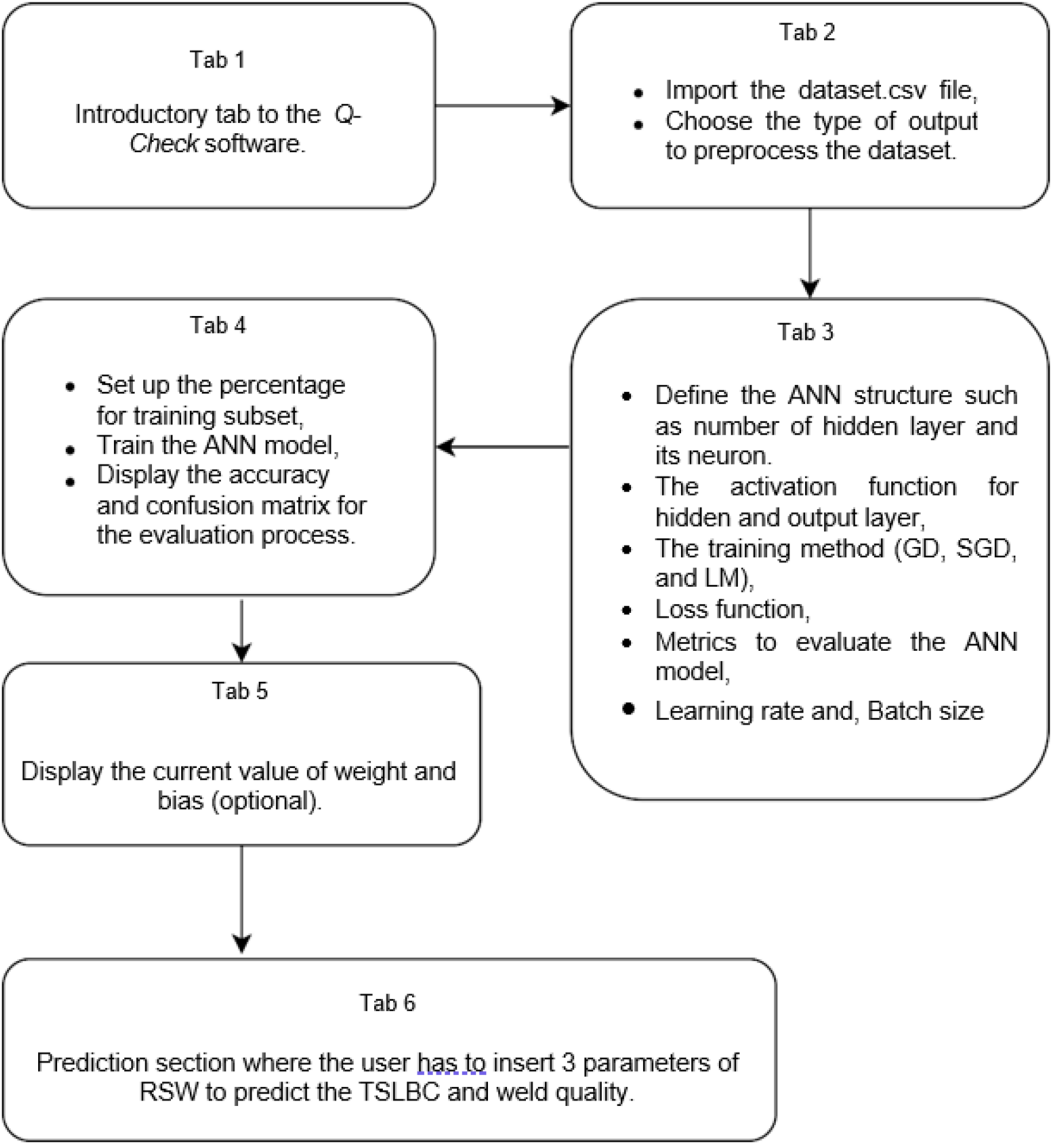
Process flow performed in the *Q-Check* application tool.

In this study, the application tool named *Q-Check* is developed using Qt designer to design the GUI layout and Spyder IDE as the back-end development. The *Q-Check* consists of 6 Tabs in which each tab has its own function to complete the prediction process. Figure 4 demonstrates the general functionalities for Tab 1 until Tab 6 in the *Q-Check* application tool.

**Figure 5:**
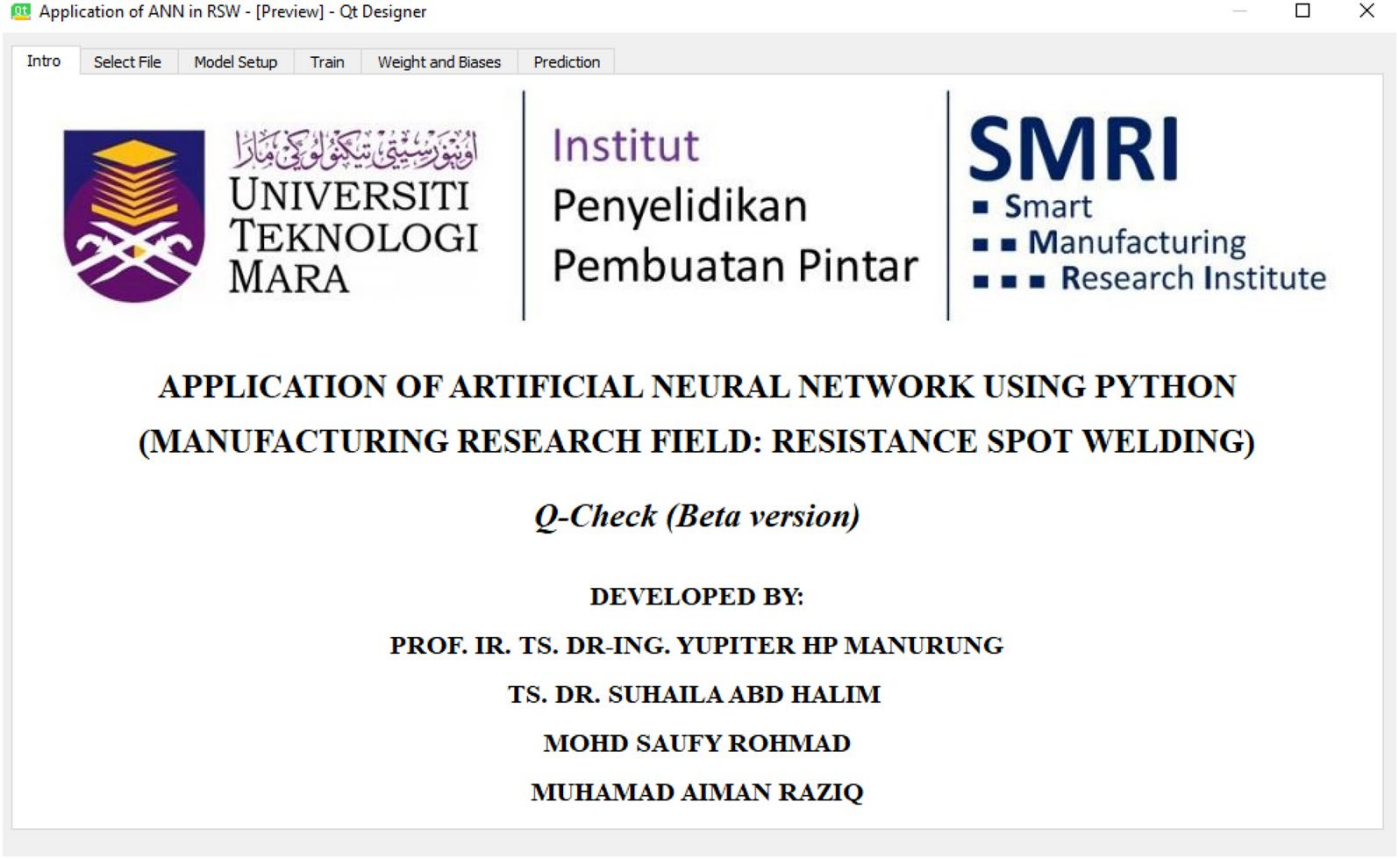
Introductory tab of *Q-Check* application tool as Tab 1.

**Figure 6:**
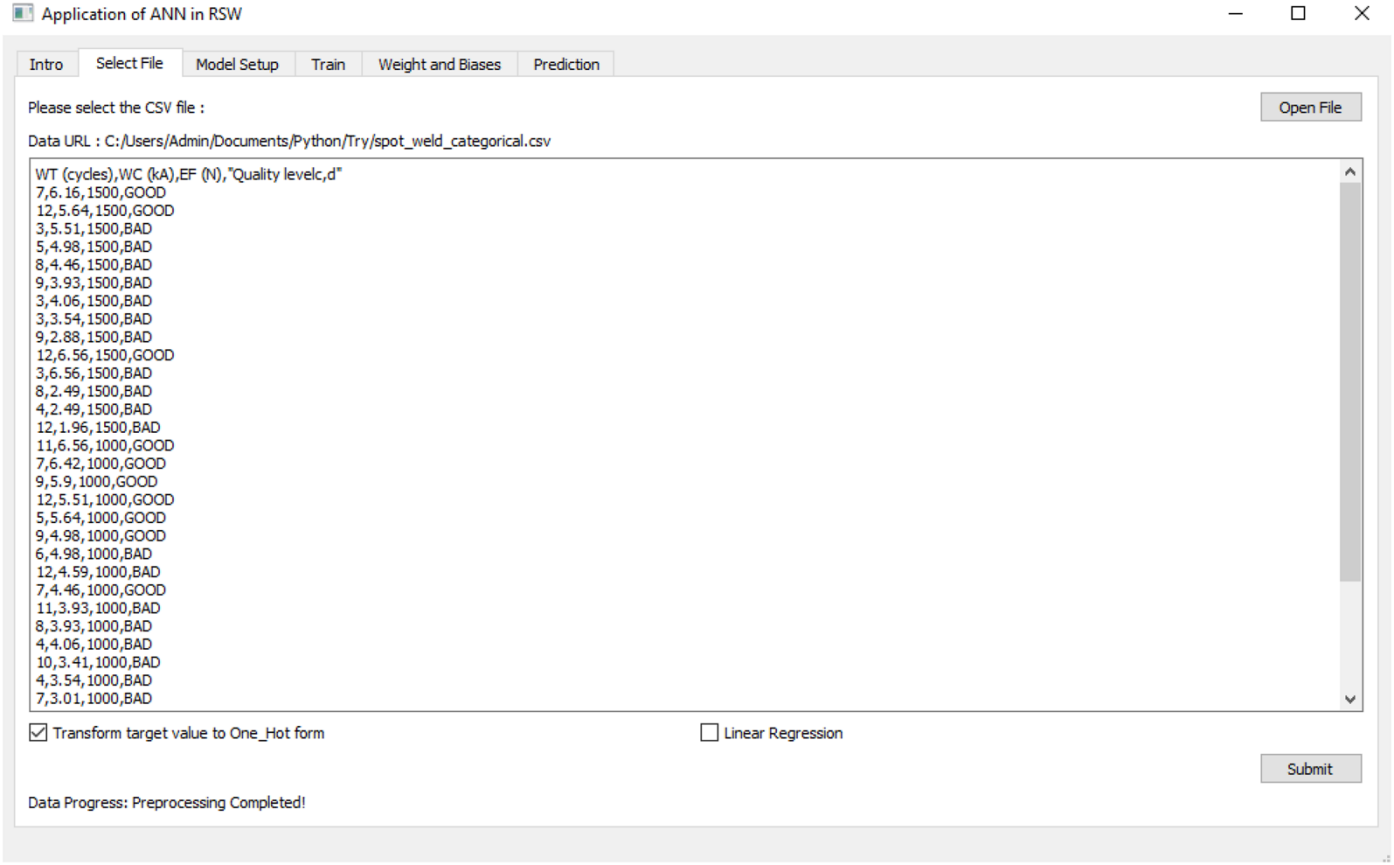
Import and pre-processing of the dataset as Tab 2.

**Figure 7:**
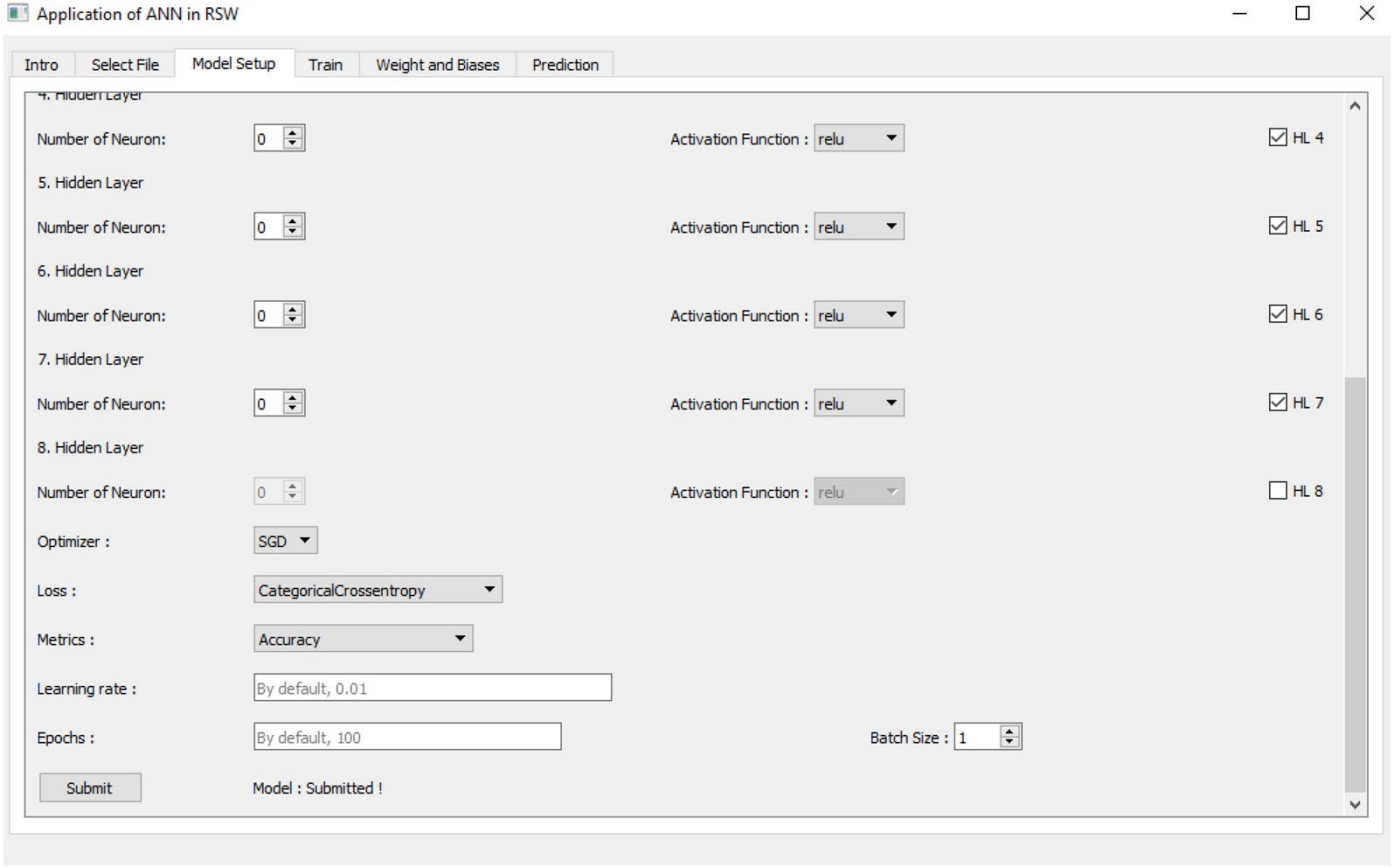
ANN modelling section as Tab 3.

**Figure 8:**
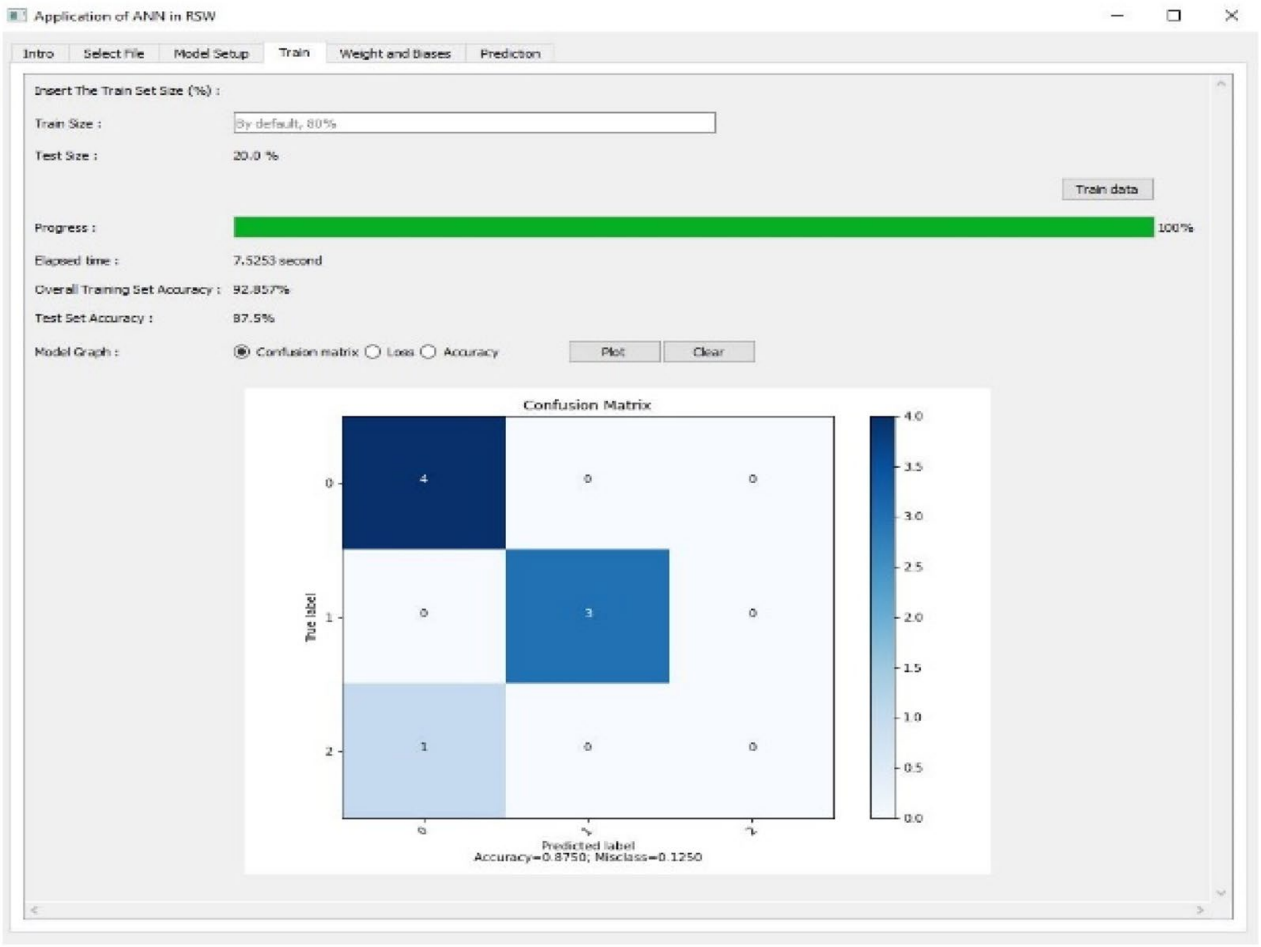
Training and evaluating the ANN model using Confusion Matrix as Tab 4.

**Figure 9:**
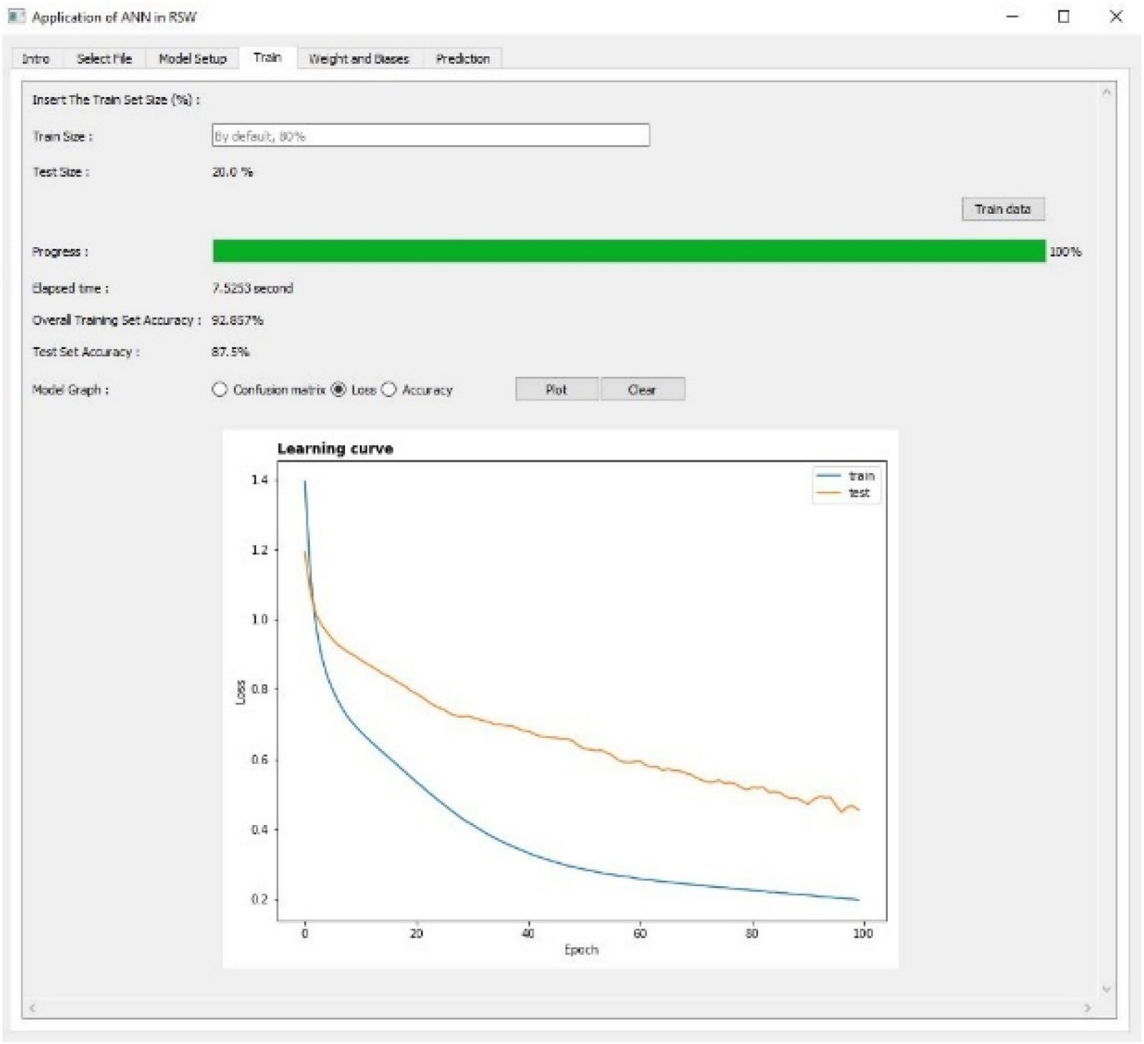
Training and evaluating the ANN model using Loss Function as Tab 4.

**Figure 10:**
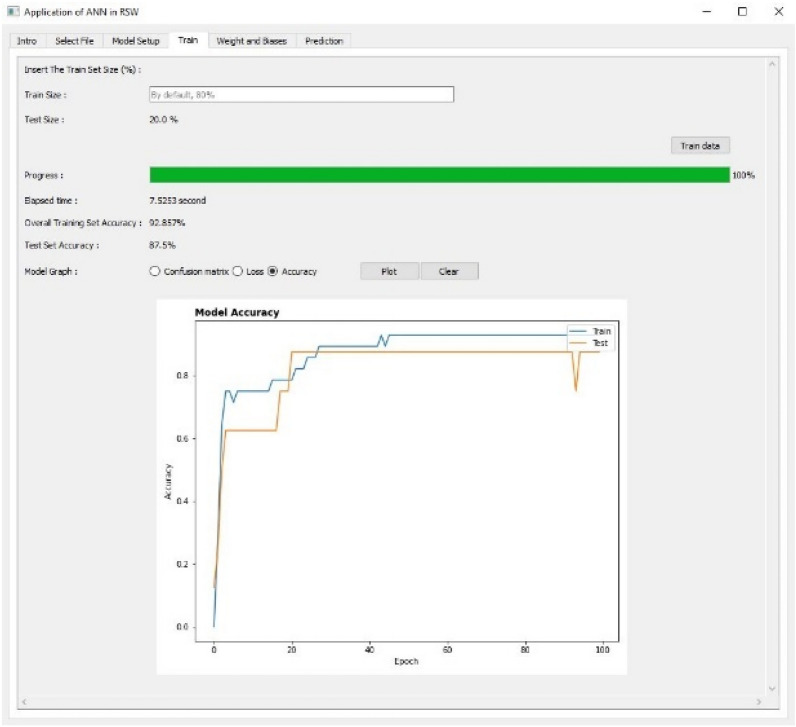
Training and evaluating the ANN model using Accuracy as Tab 4.

The layout designs of *Q-Check* with 6 tabs are shown in Figs. 5, 6, 7, 8, 9 and 10. The first tab introduces the front page of the application tools as shown in Fig. 5.

Figure 6 shows the second tab where the user is able to import the input file and pre-process dataset in the input file for further process. In pre-processing, the user can select which type of true value to be pre-processed before setting up the ANN model.

The third tab consists the setting up of the model structures and other relevant parameters to implement the ANN model. In this tab, the user is required to choose the optimizer of GD, SGD and LM training algorithm to train the model. Figure 7 shows the layout of Tab 3 for model setup.

The fourth tab as shown in Figs. 8, 9 and 10 are used to train and evaluate the performance of the ANN model using confusion matrix, loss and accuracy respectively. The training size of the dataset can be set up in this tab and the default value is 80%. The confusion matrix can be plotted for multi-classification case.

**Figure 11:**
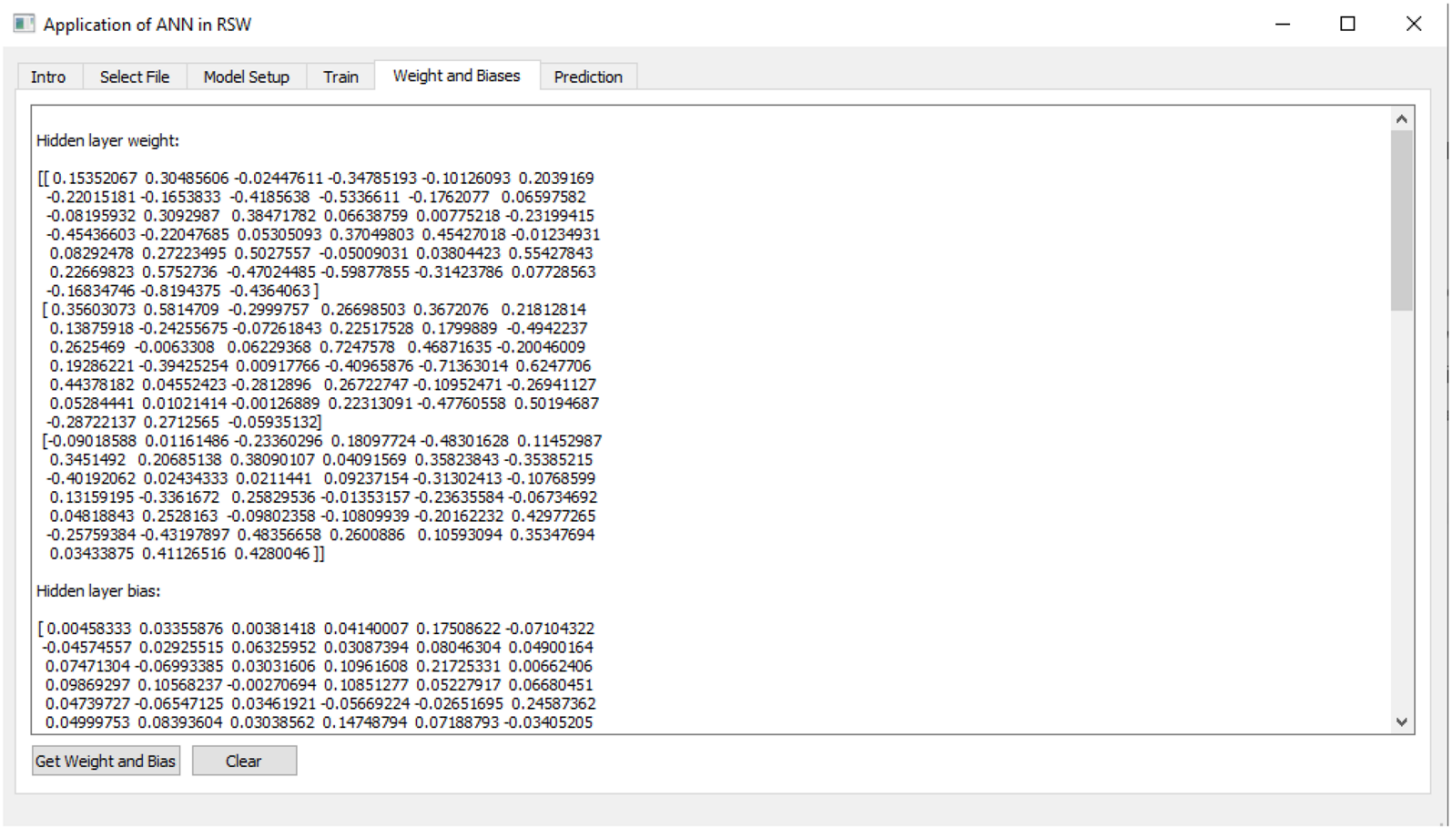
Display of current weights and biases of the ANN model as Tab 5.

The fifth tab as shown in Fig. 11 displays the current value of the weight and bias of the model. The weight and bias values for each layer of ANN model are shown and used for research purposes.

**Figure 12:**
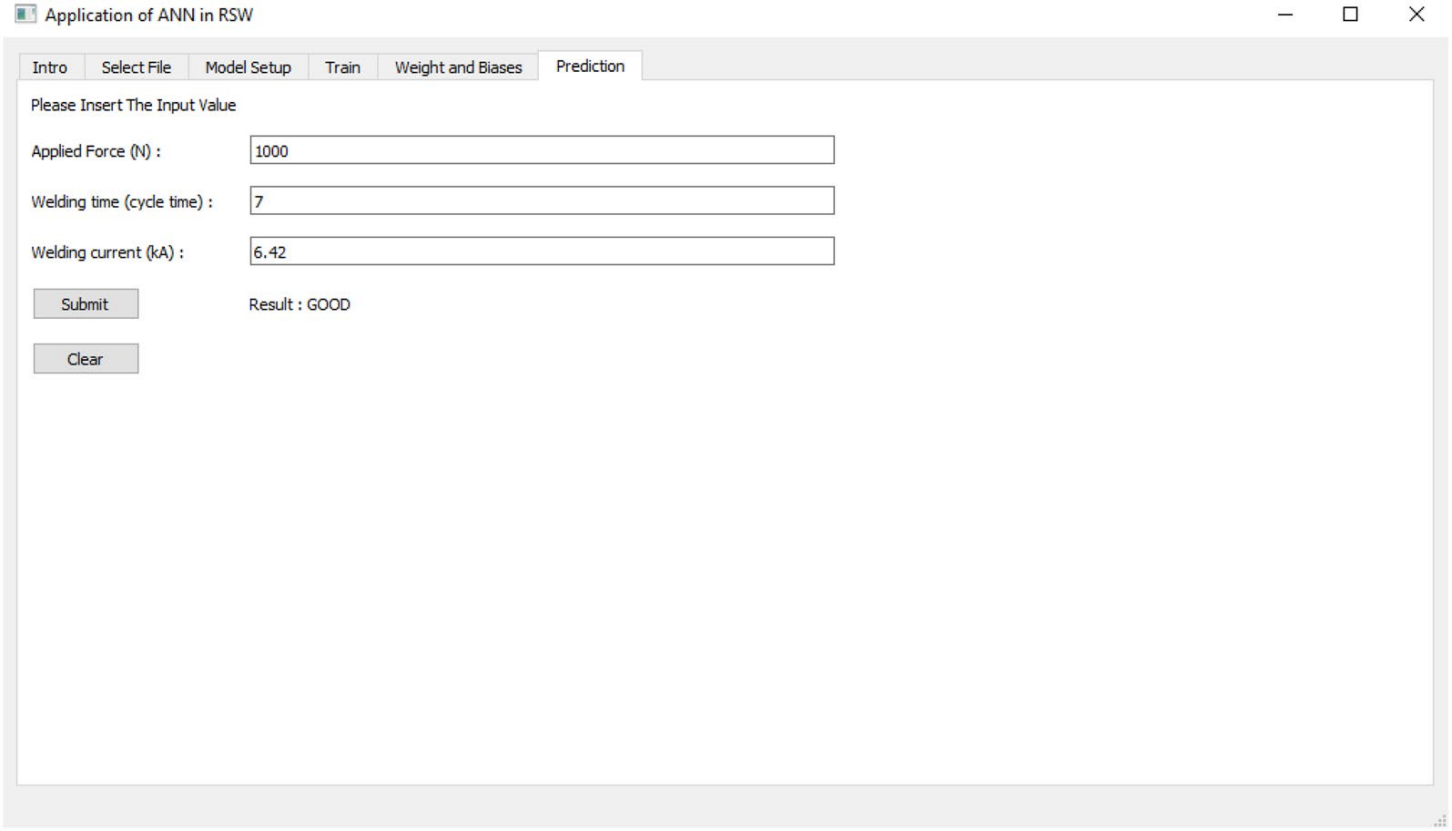
Prediction section as Tab 6.

The last tab as in Fig. 12 is used to predict the weld quality and TSLBC of RSW with the corresponding input from the user. The user is required to provide three input parameters to make the prediction based on the developed training algorithm.

## Result and discussion

The result of the research is an application tool that has the capability of evaluating and predicting the result of TSLBC value and the quality of RSW using GD, SGD and LMBP algorithms. The results obtained in this section are generated from *Q-Check* using the Qt designer from PyQt5 with Spyder IDE. During the training process, the number of epochs for the GD and SGD algorithms is 100 while the LM algorithm is 50, and the value of learning rate used is 0.01.

Table 7 shows the results of *MSE* and accuracy acquired in training and testing of GD, SGD and LM algorithms for numerical output using 2 different ANN structures.

**Table 7:** *MSE* and accuracy of the linear regression for GD, SGD and LM algorithms.

ANN structure	Training algorithm	MSE		Accuracy (%)
		Training (%)	Testing (%)	
3-39-1	GD	7.545	12.78	87.220
	SGD	1.554	7.135	92.865
	LM	2.215	6.330	93.670
3-39-39-1	GD	11.749	11.827	88.173
	SGD	1.09	10.716	89.284
	LM	1.569	6.86	93.14

The *MSE* obtained from Table 7 shows that the LM produced the highest accuracy overall while GD gave the lowest accuracy. Based on Table 7, there was a slight variance of accuracy between 1 and 2 hidden layers of ANN structure. The structure with 1 hidden layer tended to produce an average higher accuracy for all algorithms. Hence, this ANN model is used in predicting the TSLBC of RSW since it has an excellent ability to generalize the 28 input/target pairs employed during the training process. For both structures, the LM gave the highest accuracy while the LM algorithm had a faster rate convergence since it required half value of the epochs than GD and SGD algorithms.

Based on the accuracy result from Table 8, it shows that the ANN model using SGD and LM tended to generalize well for the categorical output of the RSW dataset as it provided an accuracy of 75% for both structures. The GD algorithm gave the lowest performance for both ANN structures with 62.5% of accuracy.

**Table 8:** Accuracy of the non-linear model using GD, SGD and LM algorithms.

ANN structure	Training algorithm	Train set (%)	Test set (%)
3-39-3	GD	85.714	62.5
	SGD	96.429	75.0
	LM	89.286	75.0
3-39-39-3	GD	78.571	62.5
	SGD	96.429	75.0
	LM	92.857	75.0

**Figure 13:**
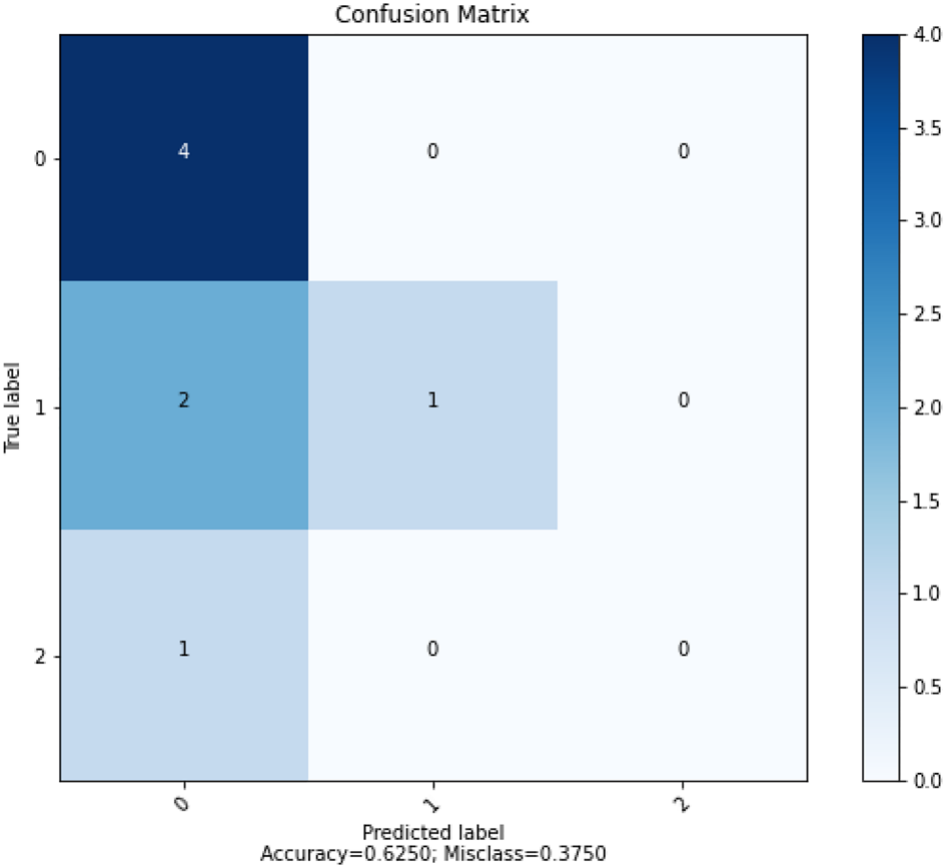
Confusion matrix for GD.

In order to summarize the performance of the ANN algorithms, confusion matrix for each algorithm was plotted. Figure 13 shows the result of confusion matrix for GD algorithm. The number 0 represents Good, 1 is Bad and 2 is Worst which refer to the RSW weld quality that has been encoded earlier.

The confusion matrix showed that the ANN model using GD algorithm correctly predicted 5 out of 8 data points.

**Figure 14:**
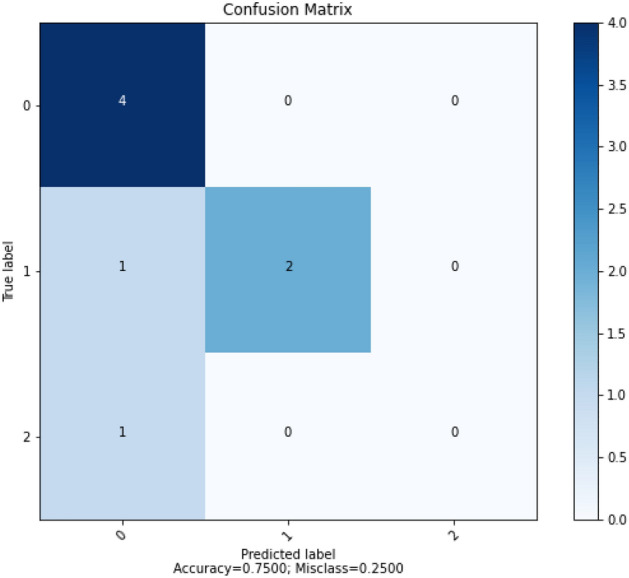
Confusion matrix for SGD.

Figure 14 shows the result of confusion matrix for SGD algorithm. The confusion matrix showed that the ANN model using SGD algorithm correctly predicted 6 out of 8 data points.

**Figure 15:**
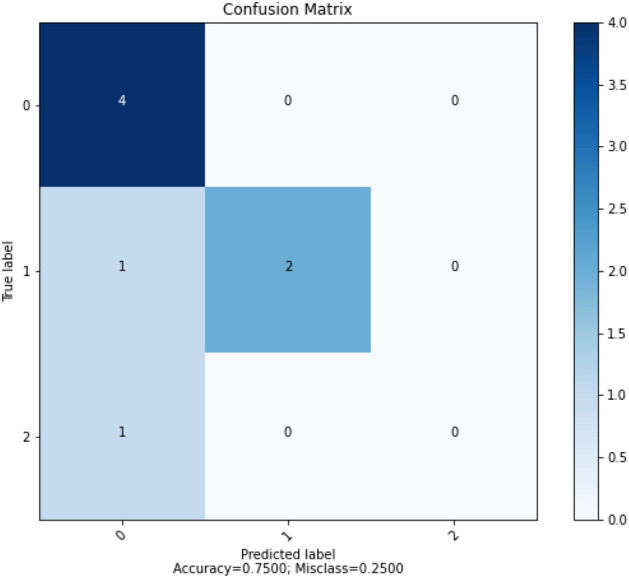
Confusion matrix for LM.

Figure 15 shows the result of confusion matrix for LM algorithm. The confusion matrix showed that the ANN model using LM algorithm correctly predicted 6 out of 8 data points.

## Conclusion and recommendation

An investigation focusing on the development of an application tool that is able to predict the TSLBC and quality weld of RSW using the ANN model was developed in this study. An application tool was developed using an open-source library and module of Tensorflow and Keras, PyQt5 and Qt designer, Matplotlib, and others. Moreover, the highlight of this study was neglecting the need for an experimental set up in calibrating RSW equipment and materials preparation. The ANN training algorithm proposed in this study was BPNN algorithm which consisted of GD, SGD, and LM. This presented a work approach in which all parameters, geometry, dimensions, and boundary conditions were set in a similar means to ensure the realistic comparison with experimental and conventional experimental study of^3^. According to the results of this study, the following conclusions can be drawn:

1. The GUI-based application tool was successfully developed in predicting the weld quality and TSLBC of RSW using Tensorflow, Keras with the Spyder IDE.
2. The accuracy produced from the prediction of the TSLBC of RSW for GD, SGD, and LM training algorithms was 82.220%, 92.865%, and 93.670%, respectively, for ANN structure 3-39-1. Meanwhile, for ANN structure of 3-39-39-1, the accuracy was 88.173%, 89.284%, and 93.140% for GD, SGD, and LM training algorithms, respectively.
3. For ANN structure of 3-39-1, the accuracy obtained from predicting the quality weld of RSW using the GD, SGD, and LM training algorithms was 62.5%, 75%, and 75%, respectively. The accuracy obtained for the 3-39-39-3 ANN structure was identical to that obtained with the 3-39-3 structure.
4. The results obtained in predicting TSLBC and the quality weld of RSW showed that the SGD and LM algorithm produced significant results to generalize the ANN model.
5. The LM algorithm had a faster rate convergence than the GD and SGD algorithms since it only used half the epoch than GD and SGD while obtaining the highest accuracy in predicting TSLBC and weld quality.
6. The proposed methodology was relatively successful and straightforward to be used in a variety of other procedures. Additionally, adopting the suggested technique enables engineers to adjust parameters directly using a GUI-based application tool without any prior theoretical understanding of neural computing.

As further recommendations to current research, the following focus can enhance the application with:

1. Adding the various types of machine learning algorithms into the application tool such as Adam, RMSProp, Adadelta and others.
2. Modification to the current GUI by adding more features such as plotting graph tools and exporting the result into excel format for reporting purposes.

## Data Availability

All data generated or analysed during this study are included in this published article.
